# Adult-Onset Acquired Ichthyosis Revealing an Underlying Colon Adenocarcinoma

**DOI:** 10.7759/cureus.73045

**Published:** 2024-11-05

**Authors:** Maria El Gemayel, Tatiana Hawat, Brian A Cahn, Umer Nadir, Michael D Yi, Maria Tsoukas, Roger Haber

**Affiliations:** 1 Gastroenterology and Hepatology, University of Illinois Chicago, Chicago, USA; 2 Dermatology, Saint George Hospital University Medical Center, Beirut, LBN; 3 Dermatology, University of Illinois Chicago, Chicago, USA; 4 Dermatology, Henry Ford Health System, Detroit, USA

**Keywords:** acquired ichthyosis, colon adenocarcinoma, ichthyosiform dermatoses, internal diseases, malignancy

## Abstract

Acquired ichthyosis is an uncommon dermatologic disorder that presents in adulthood and is often associated with systemic conditions, including malignancies. We report the case of a 38-year-old male patient who developed diffuse scaling characterized by rhomboidal, fish-like scales predominantly affecting the trunk and limb extensors, with sparing of the flexures, palms, and soles. Initial therapeutic interventions with emollients and corticosteroids were unsuccessful. A skin biopsy confirmed the diagnosis of acquired ichthyosis, and subsequent diagnostic imaging revealed an underlying colon adenocarcinoma. Notably, the patient's family history was significant for his mother's colon adenocarcinoma, suggesting a potential genetic predisposition. This case highlights the critical importance of conducting a comprehensive diagnostic evaluation for underlying malignancies upon the diagnosis of acquired ichthyosis, particularly in patients with pertinent familial cancer histories. Although the patient was lost to follow-up, this case underscores the role of acquired ichthyosis as a potential paraneoplastic marker, emphasizing the need for early detection and management of associated malignancies.

## Introduction

Ichthyosis is a large spectrum of diseases characterized by a disruption in the cornification process, leading to abnormalities in the stratum corneum barrier function and hyperkeratosis. Acquired ichthyosis is a rare subtype that presents in adulthood and has been linked to various inflammatory and neoplastic systemic diseases [1]. The pathogenesis of acquired ichthyosis involves a disruption in the normal desquamation process, leading to hyperkeratosis and scaling. Histologically, acquired ichthyosis often shows hyperkeratosis with a reduced or absent granular layer.

Acquired ichthyosis is associated with a variety of underlying diseases, including malignancies, autoimmune disorders, metabolic conditions, drug-induced reactions, and infections. The most common malignancy linked to acquired ichthyosis is Hodgkin's lymphoma. Other associated conditions include non-Hodgkin's lymphoma, multiple myeloma, and various autoimmune diseases such as systemic lupus erythematosus and Sjögren's syndrome. Endocrine disorders such as hypothyroidism and metabolic disorders like malnutrition and chronic renal failure are also implicated [2].

The paraneoplastic phenotype of acquired ichthyosis is particularly notable in its association with malignancies. Acquired ichthyosis can precede, coincide with, or follow the diagnosis of the underlying malignancy. The severity of acquired ichthyosis often correlates with the activity of the underlying disease and may improve with the treatment of the primary condition. In some cases, acquired ichthyosis can serve as a marker for disease recurrence or relapse [3].

In summary, the pathogenesis of acquired ichthyosis involves disrupted desquamation, and it is associated with a range of underlying diseases, most notably Hodgkin's lymphoma. The paraneoplastic phenotype of acquired ichthyosis can reflect the activity of the associated malignancy and may serve as a clinical marker for disease progression [4].

We report herein the case of a 38-year-old male patient with acquired ichthyosis that revealed an underlying colon adenocarcinoma.

## Case presentation

**Figure 1:**
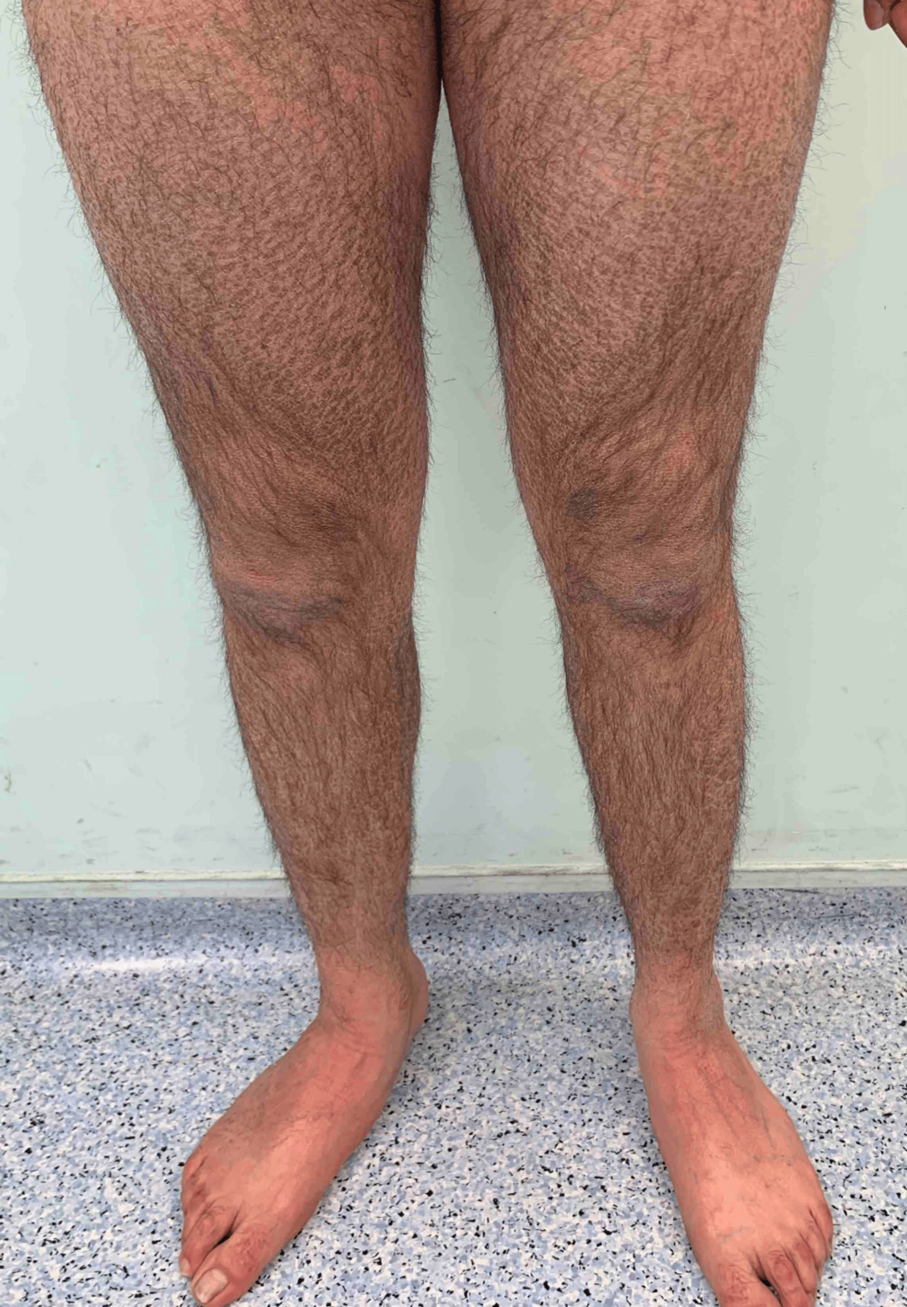
Ichthyosis affecting the patient's lower extremities

**Figure 2:**
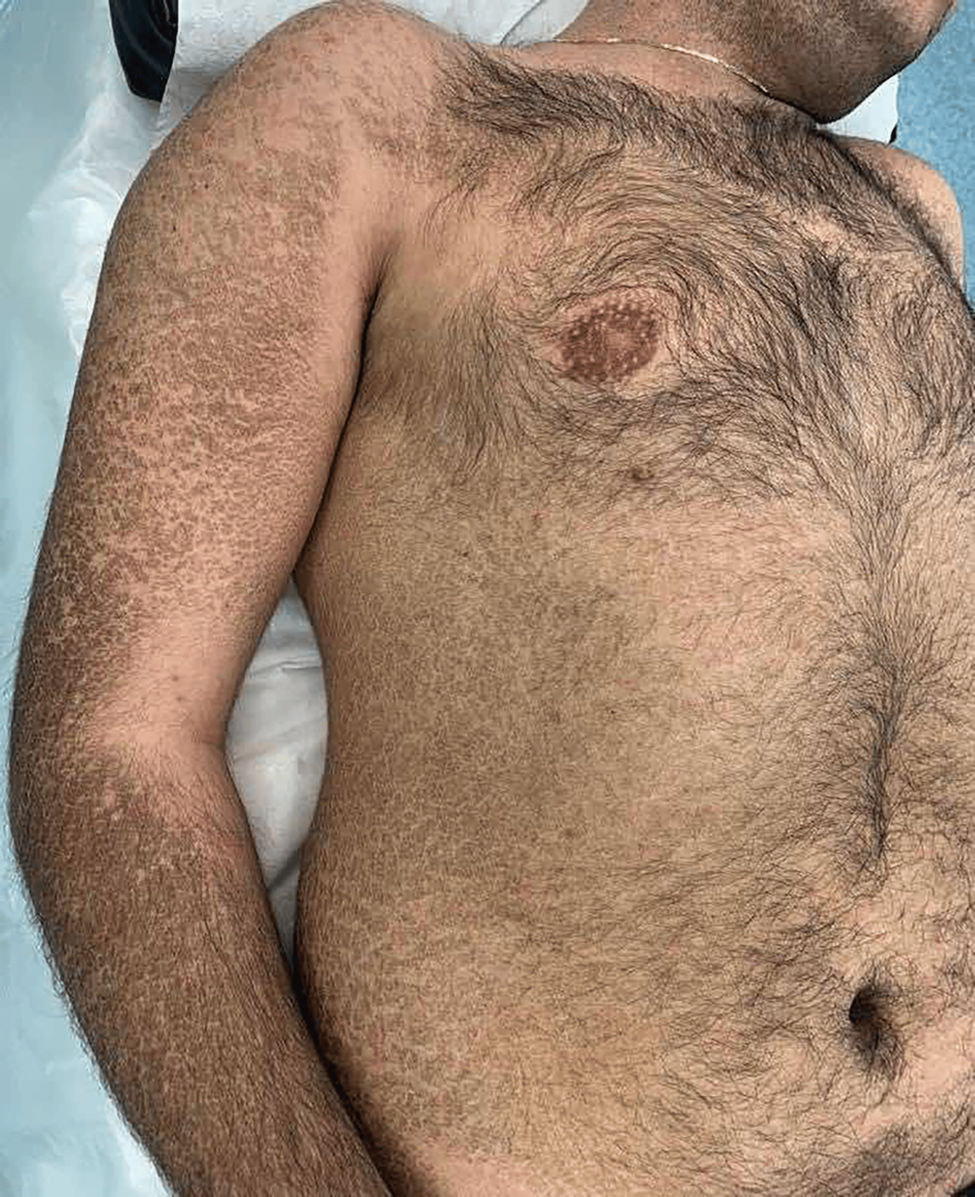
Hyperpigmented polygonal scales seen on the patient’s right upper extremity, chest, and abdomen

**Figure 3:**
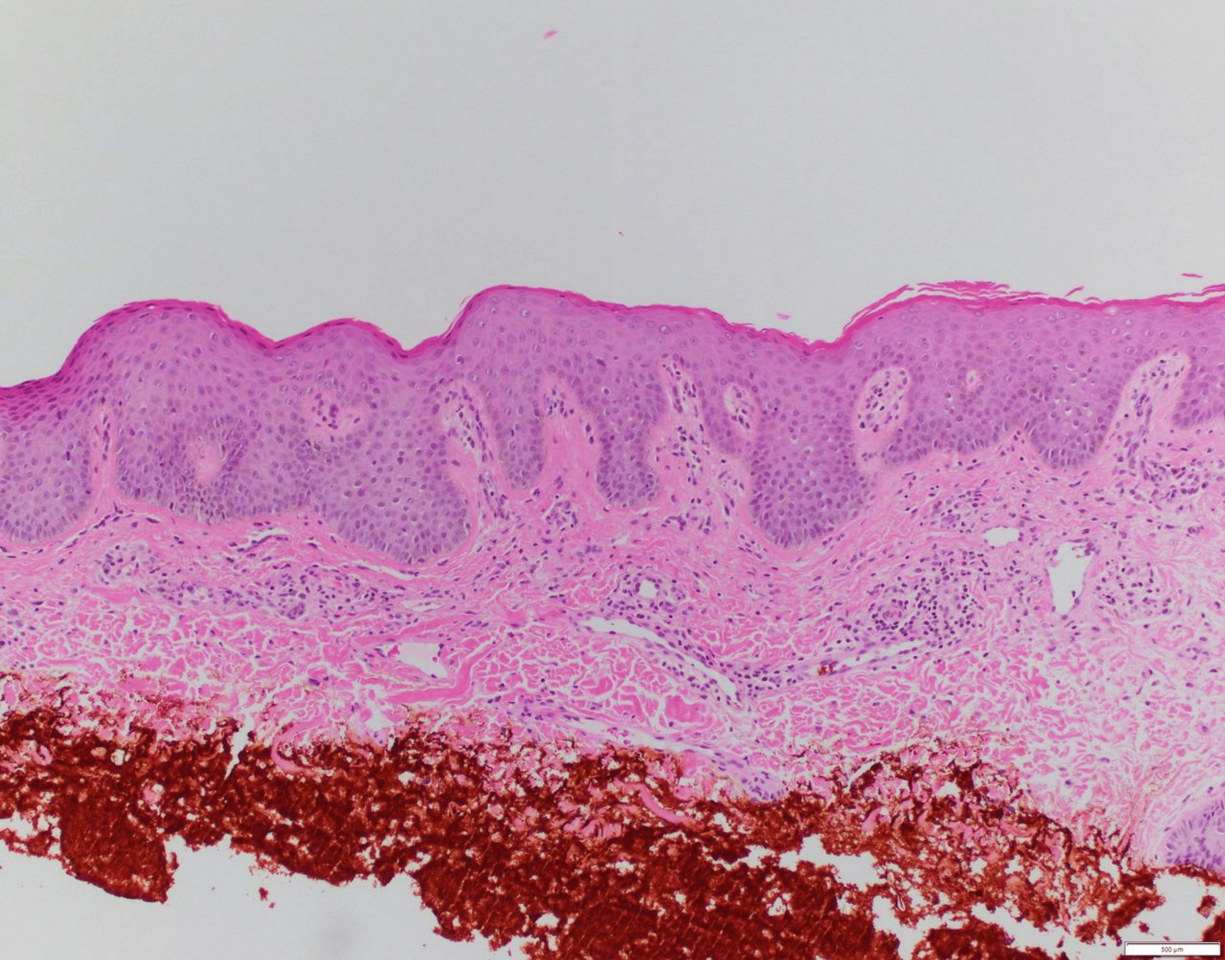
A representative histologic image of acquired ichthyosis

A 38-year-old male patient presented with a three-month history of diffuse skin scaling. The physical examination was notable for dry, rhomboidal brown scales overlying the trunk, back, and limb extensors and sparing the flexures, palms, and soles (Figures 1, 2). The patient was previously treated with emollients and topical corticosteroids with no significant improvement. He denied any new medication intake, recent travel, unprotected sexual activity, or substance use and had a normal, balanced diet. Family history revealed the presence of adenocarcinoma of the colon in his mother. Liver, renal, endocrine, and hormonal laboratory values were all within normal limits. Due to a lack of improvement in emollients and corticosteroids, ichthyosis vulgaris and xerosis were excluded. Additionally, the lack of associated neurologic findings excluded Refsum disease. The diagnosis of adult-onset acquired ichthyosis was made clinically and confirmed by skin histology that showed compact orthokeratosis of the cornified layer and thinning of the granular layer with no granuloma or atypical cells (Figure 3) [5].

**Figure 4:**
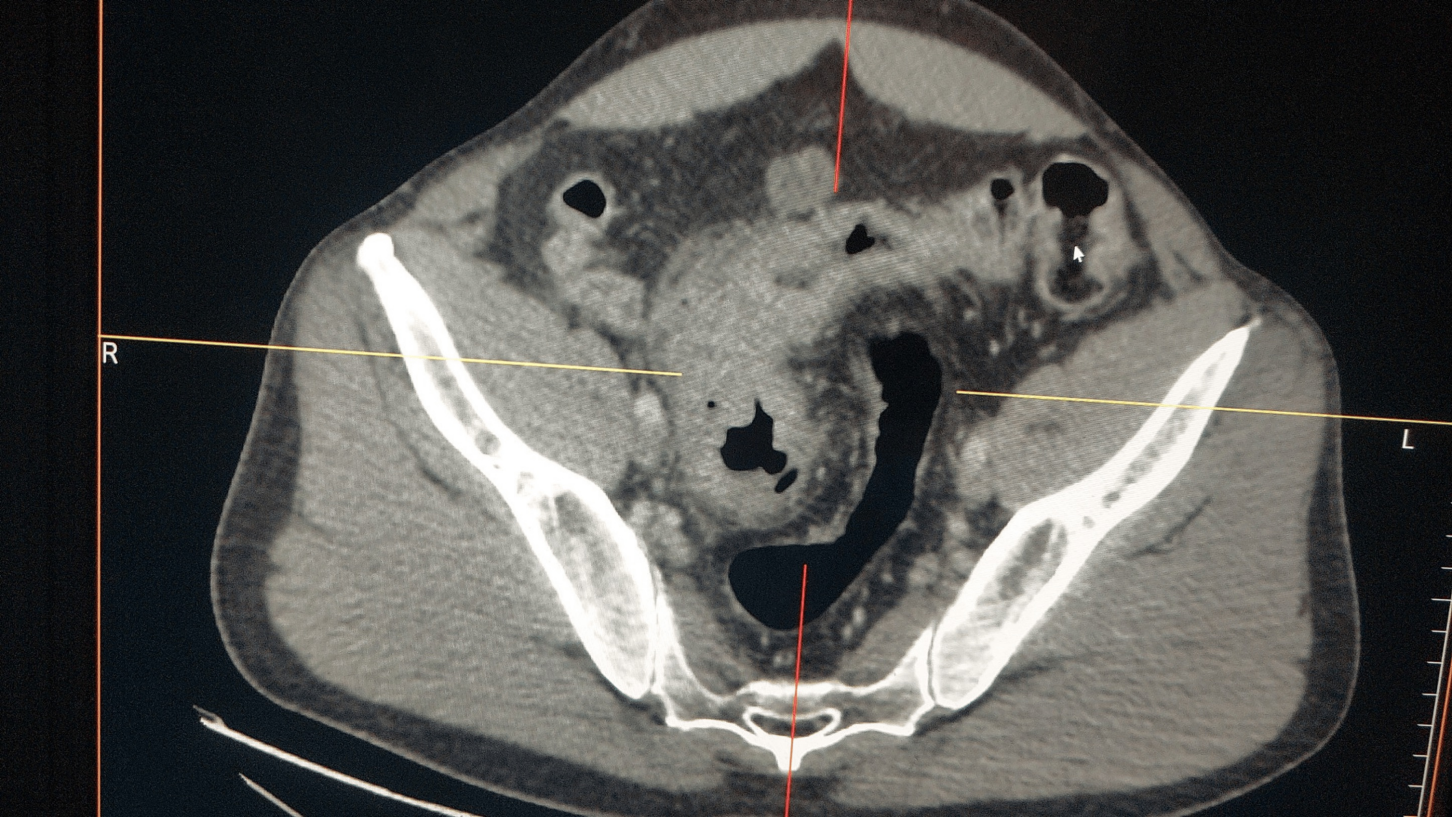
Computed tomography scan revealing an adenocarcinoma of the colon

A total body computerized tomography scan during workup three months after symptom onset revealed a 2-cm polypoid tumor of the ascending colon, and a colonoscopy confirmed the diagnosis of colon adenocarcinoma with tissue biopsies (Figure 4). The patient was then lost to follow-up.

## Discussion

Adult-onset acquired ichthyosis is a nonhereditary rare skin disease characterized by symmetric fish-like scaling mainly on the trunk and limb extensors. Associations with malignancies (Hodgkin's lymphoma, hematologic malignancies, and visceral carcinomas), inflammatory (systemic lupus erythematosus, dermatomyositis), autoimmune (eosinophilic fasciitis), metabolic (chronic hepatic or renal failure), endocrine (hypothyroidism, hyperparathyroidism), malabsorption, drug-induced, and infectious diseases (acquired immunodeficiency syndrome, human T-lymphotropic virus, leprosy) have been sparsely reported [1]. To our knowledge, this is one of the first cases of acquired ichthyosis revealing an underlying colon adenocarcinoma.

In our patient, given the acute onset of symptoms, the lack of improvement with topical emollients and topical corticosteroids, the histologic evidence, and a confirmed diagnosis of colon adenocarcinoma, a diagnosis of acquired ichthyosis was made. Once the diagnosis of acquired ichthyosis is made, the focus should be on finding the underlying cause through a complete history (recent travel, dietary routine, drug intake, and detailed system review) and a full clinical examination. Skin histology is helpful to exclude other disorders, such as cutaneous disorders, as the differential diagnosis includes those with a reduced or absent granular layer. Important exclusions include sarcoidosis, where caveating granulomas are seen [3]; cutaneous T-cell lymphoma, where atypical cells [6] are seen; asteatotic dermatitis, where an inflammatory infiltrate is seen; mycosis fungoides, where epidermotropism of atypical lymphocytes is seen; and psoriasis, where Munro microabscesses and other inflammatory infiltrates are seen.

Additional tests include a complete blood count with differential, complete metabolic profile, thyroid function tests, serum protein electrophoresis, immunofixation electrophoresis, an autoimmune panel including ANA (antinuclear antibody), rheumatoid factor, anti-dsDNA, HIV testing, vitamin D level, sexually transmitted infection swabs, and if lymphadenopathy is present, lymph node biopsy. If the workup is largely negative, consider a full-body computerized tomography scan to rule out malignancy. The most commonly reported malignancy in association with adult-onset acquired ichthyosis is Hodgkin's disease (in up to 80% of cases) [2]. However, associations with non-Hodgkin's lymphoma, leiomyosarcoma, mycosis fungoides, multiple myeloma, Kaposi's sarcoma, lymphomatoid papulosis, and carcinomas of the ovary, breast, gastric, lung, and cervix were also reported [7]. The mechanisms of these associations have been described above.

A limitation of our case is that there was a lack of follow-up, which the authors acknowledge may result in a lack of a critical variable regarding the improvement of ichthyosis following tumor treatment.

## Conclusions

Acquired ichthyosis may occur simultaneously with or subsequent to the diagnosis of malignancy and often resolves upon treatment of the underlying malignancy, possibly serving as a marker of therapeutic response or disease recurrence. In our case, the diagnosis of an underlying colon adenocarcinoma was confirmed with a colonic tissue biopsy during colonoscopy three months after the onset of acquired ichthyosis, highlighting the importance of a thorough workup for underlying malignancy once the diagnosis is made. The presence of a positive family history of colon cancer in his mother could also be linked to a genetic component. The management of acquired ichthyosis is challenging, and the goal should not only be focused on relieving skin symptoms but also on identifying and treating the underlying etiology.
